# The Science of Harmony: A Psychophysical Basis for Perceptual Tensions and Resolutions in Music

**DOI:** 10.34133/2019/2369041

**Published:** 2019-09-29

**Authors:** Paul Yaozhu Chan, Minghui Dong, Haizhou Li

**Affiliations:** ^1^Institute for Infocomm Research, A⁎STAR, Singapore; ^2^Nanyang Technological University, Singapore; ^3^National University of Singapore, Singapore

## Abstract

This paper attempts to establish a psychophysical basis for both stationary (tension in chord sonorities) and transitional (resolution in chord progressions) harmony. Harmony studies the phenomenon of combining notes in music to produce a pleasing effect greater than the sum of its parts. Being both aesthetic and mathematical in nature, it has baffled some of the brightest minds in physics and mathematics for centuries. With stationary harmony acoustics, traditional theories explaining consonances and dissonances that have been widely accepted are centred around two schools: rational relationships (commonly credited to Pythagoras) and Helmholtz's beating frequencies. The first is more of an attribution than a psychoacoustic explanation while electrophysiological (amongst other) discrepancies with the second still remain disputed. Transitional harmony, on the other hand, is a more complex problem that has remained largely elusive to acoustic science even today. In order to address both stationary and transitional harmony, we first propose the notion of interharmonic and subharmonic modulations to address the summation of adjacent and distant sinusoids in a chord. Based on this, earlier parts of this paper then bridges the two schools and shows how they stem from a single equation. Later parts of the paper focuses on subharmonic modulations to explain aspects of harmony that interharmonic modulations cannot. Introducing the concept of stationary and transitional subharmonic tensions, we show how it can explain perceptual concepts such as tension in stationary harmony and resolution in transitional harmony, by which we also address the five fundamental questions of psychoacoustic harmony such as why the pleasing effect of harmony is greater than that of the sum of its parts. Finally, strong correlations with traditional music theory and perception statistics affirm our theory with stationary and transitional harmony.

## 1. Introduction

Even though it is one of the most important components in music, and possibly the most widely studied [1], the definition of harmony differs vastly across time, genre, and individuals, reflecting how little is understood about it [2, 3].

There are three aspects to the complete understanding of our perception of harmony, which we will, for brevity, refer to as* what*,* why*, and* when*. The* what* of harmony refers to an attribution to a defining quality. Its* why* goes further to explain the means by which such a quality ascribes to consonance or dissonance (or even sentiment or emotions). Finally, it should be recognized that the same harmony perceived as consonant in one context can be perceived as dissonant in another. This takes the* what* and* why* of stationary harmony (sonorities) into the context of transitional harmony (progression). We refer to this as the* when* of harmony and it has remained largely unaddressed by acoustic science.

### 1.1. Background

Early works effectively attributed the* what* of harmony to rational relationships [1, 4]. This ascribes a chord's consonance to the ratio amongst its contributing string lengths (and consequently, wave periods and fundamental frequencies), being fractional with integer numerators and denominators. A fascinating number of esteemed mathematicians, physicists, and philosophers have made different contributions in this aspect. The development of the Pythagorean tuning system is commonly credited to Pythagoras in the fourth century BC [3, 5, 6]. Euclid wrote the earliest surviving record on the tuning of the monochord [7] and documented numerous experiments on rational tuning [8]. Aristotle and Plato made various contributions to the development of ancient Grecian (rationally scaled) music that was later integrated into the diatonic system [8, 9]. Ptolemy developed the syntonic diatonic system as early as the second century [10]. Euler proposed a grading system of chord aesthetics based on the assertion that the notes have a least common multiple (i.e., that they are rational) [11]. Since string lengths correspond to wavelengths, which correspond to wave period, and since notes used in harmony are taken from the scale, it can be said that the Pythagorean school effectively attributes harmony to temporal features.

It was not until 1877 that Helmholtz pioneered the psychoacoustic approach [3, 8, 12, 13]. Isolating adjacent harmonic sinusoids from different notes using specifically devised acoustic resonators, he was able to record how amplitude modulation that resulted from their summation grew perceptually unpleasant as their modulation frequency increased towards a certain threshold [8], thus attributing dissonance to what he called beating frequencies and addressing the questions* what sounds bad and why*. Numerous others [14–25] conducted further studies in this approach, while others raised several questions with Helmholtz's theory [13, 17, 26]. For example, Plomp and Levelt [12] and Schellenberg and Trehub [27] have separately shown that consonances and dissonances are still perceived in harmonies with pure tones (tones without harmonics). Itoh [28] and Bidelman [29], amongst others, also showed that electrophysiological responses to pure-tone intervals did not agree with Helmholtz. All in all, the Helmholtz school attributes harmony to frequency features and comprises a large part of what is referred to in this paper as interharmonic modulations.

In 1898, a notable but short-lived [3] attempt at* what sounds good and why* was seen in Stumpf's tonal fusion theory [30], which theorized that harmony was the effect the harmonics of its component notes fusing together to sound like a single note with a common fundament [12, 13, 26, 30].

Because of the nonlinear relationship between tonal scale and frequency, scales derived from rational lengths of a string tended to leave certain intervals more rational than others. With this realization, Western music eventually adopted 12-tone equal temperament scale. This equally segments the octave in the log-frequency scale [31] such that each semitone interval is a factor of 2^1/12^, evenly redistributing the dissonances to accommodate to different keys. Despite its late adoption, original development of this scale predates Helmholtz to the 1500s. Vincenzo Galilei (father of Galileo Galilei) made the earliest known estimate of this in the West by approximating 2^1/12^ with 18/17 [32], while Zhu was credited for perfecting it in the East by computing it to accurately to the 25^th^ decimal, both in the 1580s [12]. The earliest recorded estimate of this in the East was by He in the 5th century, whose estimate was already about as accurate as Galilei's [33, 34].

In Rameau's Treatise on Harmony [1], which paved the foundations of harmony in modern music theory, notes of basic chords are derived from the division of the length of a common string [35]. However, this remains disjoint with the rest of the treatise, and modern music theory remains more of a compilation of rules and deductions from the pattern clustering of perceptual experiences [36–42], addressing the questions* what sounds good and when* without the scientific reasoning of* why *[37].

More recently, several studies have found high correlations between harmony and periodicity measures of the resultant signal [43, 44]. This novel leap advances the Pythagorean school while presenting a persuasive attribute of* what sounds good and why*.

Several notable studies have also been conducted that relate harmony to nonacoustic attributes such as statistics and geometry. An example is Tymoczko's exploration of how multidimensional geometric patterns correlate strongly with patterns that exist in historic harmony use, addressing* what sounds good and when* [45–47]. Authors in [48] explored properties of musical scales on the Euler lattice, addressing the* what* of harmony. Numerous others such as [49–51] have worked on other mathematical relationships in harmony, addressing its* what*.

Yet others have looked towards a biological rationale towards our perception of harmony to address* what sounds good and why*. A recent example is Purves' attribution of the effect of the tonal scale to the familiarity of excited or subdued speech [14, 52–54]. Other examples are the works of [43, 55, 56] in the neuronal mechanism of harmony perception.

### 1.2. Scope

In this work, we first seek a mathematical resolution across both acoustic schools by a single psychophysical theory. To start off somewhere familiar, we first describe the concept of* interharmonic modulations* (which adopts and encompasses Helmholtz's beating frequencies), from which we then introduce the concept of* subharmonic *[57]* modulations* and show how the two categories of modulations relate. (At some point after which, we also show how a specific case of subharmonic modulations addresses Pythagoras, thus integrating the two schools.) After explaining how perceptual tensions [18, 36, 58, 59] in musical harmony may be identified in subharmonic tension in the stationary context, we continue to explain how perceptual tension resolutions [18, 42] in transitional harmony (chord progressions) may be visualized in subharmonic trajectories. By these, we address the* what*,* why*, and* when* of harmony. Numerical results show strong to near-complete correlations with perception and chord-use statistics that are presented towards the end of the paper.

By applying our theory and equations, we will answer the five fundamental questions of psychoacoustic harmony. These are as follows.

(1) The phenomenon that the effect of harmony is greater than the sum of its parts [18, 60]:
  (1) $$\begin{array}{c} \varepsilon \left\{x_{1}+x_{2}+x_{3}\right\}\gg \varepsilon \left\{x_{1}\right\}+\varepsilon \left\{x_{2}\right\}+\varepsilon \left\{x_{3}\right\} \end{array}$$
where *ε* denotes* the harmonious effect of x*_1_, *x*_2_, and *x*_3_ representing notes of the chord and ‘+' denotes simultaneous presentation or cumulation.
(2) There are the definition and explanation of stationary harmony, i.e.,* what sounds good* and* why*, or, mathematically, to quantify *ε*{*X*_*n*_}, where *X*_*n*_ represents chord *n*.
(3) There are the definition and explanation of transitional harmony, i.e.,* what sounds good*,* why*, and* when*, or, mathematically, to quantify *ε*{*X*_1_ → *X*_2_}, where ‘→' denotes transition from one chord to another.
(4) We have the following phenomena.
  (a) A chord that sounds better than another out of context can sound worse than being in context [42]. Given *ε*{*X*_2_} > *ε*{*X*_3_} this shows that *ε*{*X*_1_ → *X*_2_} < *ε*{*X*_1_ → *X*_3_}
  (b) A chord that sounds better than another in one context can sound worse than being in another context [42]. Given *ε*{*X*_4_ → *X*_2_} > *ε*{*X*_4_ → *X*_3_} this shows that *ε*{*X*_1_ → *X*_2_} < *ε*{*X*_1_ → *X*_3_}
(5) We have the phenomenon that the transition from a low-tension chord to a high-tension one can still bring about the effect of tension release (resolution). Given *ε*{*X*_1_} < *ε*{*X*_2_} this shows that *ε*{*X*_1_ → *X*_2_} > 0

Apart from Pythagoras [3, 5, 6] and Helmholtz [8], we will, in closing, also briefly explain how our theory mathematically bridges other subsidiary psychophysical theories such as Stumpf [30], Euler [11], Galilei [33, 34, 61], and Zhu [12].

## 2. A Universal Theory of Harmony

In this section, a psychophysical basis for harmony is proposed as follows.

The human perception of harmony is composed of auditory events produced by the combination of sinusoids that make up each note in the harmony. These may be classified into interharmonic and subharmonic modulations.

First-order interharmonic modulations are those produced by the interplay amongst adjacent sinusoids across differing notes. These are loosely categorized by the frequency of the resultant amplitude modulation into dissonant beating frequencies [8] and consonant low-frequency modulations, triggering a variety of emotions according to their modulation and carrier frequencies. Second-order interharmonic modulations are produced by the alignment of first-order ones. The consonance types of different intervals may be identified according to patterns cast by interharmonic modulations on the interharmonic plot.

Despite the significance of interharmonic modulations, the effect of consonances and dissonances is still experienced in the absence of harmonics with pure tone harmonies. This implies that interharmonic modulations are not exclusive in our perception of harmony [12, 13, 17, 26–29]. From this, it may be deduced that subharmonic modulations also play a significant role.

Subharmonic modulations are produced by the interplay of sinusoids much further apart than interharmonic modulations. Unlike interharmonic modulations, which are analysed primarily in the frequency domain, subharmonic modulations are analysed primarily in the temporal domain and they are comprised of two parts. The first part is subharmonic wave formation, which occurs with the summation of component waveforms from each note to produce a waveform largely periodic to a common subharmonic frequency. The second is subharmonic wave deformation (an example is provided in Supplementary [Supplementary-material supplementary-material-1].), which is a distortion to every successive period of this composite subharmonic waveform due to the imperfect alignment of contributing wave periods. Stationary tension and transitional resolution may both be derived from subharmonic features which serve as measures of stationary and transitional harmony.

In order to explain interharmonic and subharmonic modulations in detail and how they unify the two prevailing schools of harmony, we will start from first principle by looking at the notes of a chord as the sum of their composite sinusoids.

### 2.1. Modulations in Sinusoidal Summation

When waveforms of two notes, *x*_1_(*t*) and *x*_2_(*t*), at amplitudes *α* and *β*, respectively, are presented together, the result may be expressed as a sum of their composite sinusoids such that

(2) $$\begin{array}{c} \alpha x_{1}\left(t\right)+\beta x_{2}\left(t\right)=\alpha \overset{N}{\underset{n=1}{\sum }}q_{n}\cos\left(2\pi nf_{1}t+\rho_{n}\right)+\beta \overset{M}{\underset{m=1}{\sum }}r_{m}\cos\left(2\pi mf_{2}t+\varphi_{m}\right) \end{array}$$

where, respectively, *n* and *m* represent the individual harmonics from each note, *N* and *M* represent the highest harmonics that need to be considered because of audible range, *q*_*n*_ and *r*_*m*_ represent the amplitude coefficients of each harmonic, *nf*_1_ and *mf*_2_ represent the frequencies of each harmonic with *f*_1_ and *f*_2_ representing the fundamental frequency of each note, *ρ*_*n*_ and *φ*_*m*_ represent the starting phases of each harmonic, and *t* represents monotonically increasing time.

Isolating a single pair of adjacent sinusoids from differing notes we get

(3) $$\begin{array}{c} Ah_{1}\left(t\right)+Bh_{2}\left(t\right)=A\cos\omega_{1}t+B\cos\omega_{2}t \end{array}$$

where *h*_1_(*t*) and *h*_2_(*t*) are the pair of harmonics from differing notes, *A* = *αq*_*n*_, *B* = *βr*_*m*_, *ω*_1_ = 2*πnf*_1_, and *ω*_2_ = 2*πmf*_2_.

Since we are considering the modulating frequency resultant of the summation of both sinusoids spanning all phase combinations, it no longer matters which starting phase we take reference from. Hence, *ρ*_*n*_ and *φ*_*m*_ can both be set to zero.

**Figure 1:**
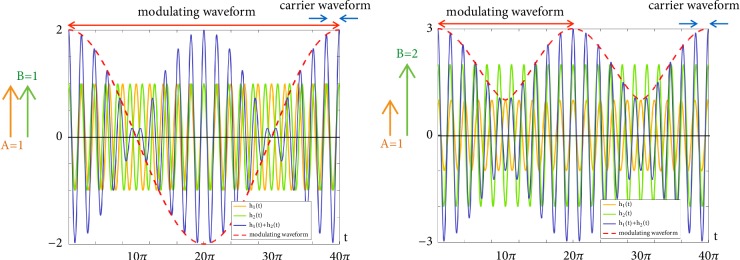
Summation of sinusoids of equal (left) versus unequal (right) amplitudes. Notice the difference in modulating frequencies even though frequencies of component sinusoids remain unchanged.

In the case of A=B, the resultant amplitude modulation is trivial and, as illustrated in Figure 1 (left), is given by the sum-to-product rule

(4) $$\begin{array}{c} \cos\omega_{1}t+\cos\omega_{2}t=2\cos\frac{∆\omega }{2}t\cos\overset{-}{\omega }t \end{array}$$

where ∆*ω*/2 is the normalized modulating frequency and is given by

(5) $$\begin{array}{c} \frac{∆\omega }{2}=\frac{\left|\omega_{1}-\omega_{2}\right|}{2} \end{array}$$

$\overset{-}{\omega }$ is the normalized carrier frequency given by

(6) $$\begin{array}{c} \overset{-}{\omega }=\frac{\omega_{1}+\omega_{2}}{2} \end{array}$$

and the values of A and B are normalized to 1.

However, in most cases, *A* ≠ *B*, and the problem becomes nontrivial, because of the change in modulation frequency as the modulating waveform no longer crosses zero. This can be seen in Figure 1 (right).

We approximate the summation of these sinusoids to be

(7) $$\begin{array}{c} A\cos\omega_{1}t+B\cos\omega_{2}t\approx B-A+2A\left\|\cos^{2-A/B}\frac{∆\omega }{2}t\right\|\cos\omega_{c}t \end{array}$$

where *ω*_*c*_ is bounded by *ω*_1_ and *ω*_2_ and is approximated to be $\overset{-}{\omega }$ (which denormalizes to $\overset{-}{f})$; ‖cos^2−*A*/*B*^⁡(∆*ω*/2)*t*‖ denotes the magnitude of cos^2−*A*/*B*^⁡(∆*ω*/2)*t* signed according to the quadrant of (∆*ω*/2)*t*. *B* denotes the larger of the amplitudes and *A* and *B* are normalized to *A* = 1.

When *A* = *B*, this simplifies to (4), where the modulating frequency is ∆*ω*/2.

However, as *B* increases with respect to *A*, 2 − *A*/*B* gravitates towards 2, and

(8) $$\begin{array}{c} A\cos\omega_{1}t+B\cos\omega_{2}t\approx B-A+2A\;\cos^{2}\frac{∆\omega }{2}t\cos\omega_{c}t\approx B+A\cos∆\omega t\cos\omega_{c}t \end{array}$$

for which the modulating frequency is ∆*ω*.

We can see from the plots in Supplementary [Supplementary-material supplementary-material-1] that this estimation is accurate for values of B marginally larger than A to much larger than A.

For consistency, the effective modulating frequency for the case of *A* = *B* will be considered by the frequency of its rectified modulating waveform which is then, similarly, ∆*ω*. In music, we are interested in this frequency in hertz. Hence, we denormalize this to be

(9) $$\begin{array}{c} \Delta f=\left|f_{1}-f_{2}\right| \end{array}$$

In the next two sections, we will move on to see how this is applicable not only to the summation of adjacent harmonics in interharmonic modulations but also to distant sinusoids in subharmonic modulations.

## 3. Interharmonic Modulations

Interharmonic modulation refers to modulations across adjacent pairs of sinusoids from different notes that fall within a certain threshold, with modulation frequency corresponding to ∆*f* in (9).

**Figure 2:**
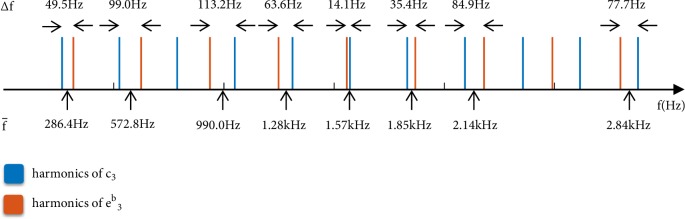
Identifying the interharmonic modulations across c_3_ and e^b^_3_.

Figure 2 shows a plot of all harmonics of notes c_3_ (blue) and e^b^_3_ (red) under 3 kHz. All adjacent sinusoids less than 120 Hz apart are identified in the figure, with their centre, $\overset{-}{f}$, and modulating, Δ*f*, frequencies labeled accordingly.

### 3.1. Beating Frequencies and Low-Frequency Modulations

**Figure 3:**
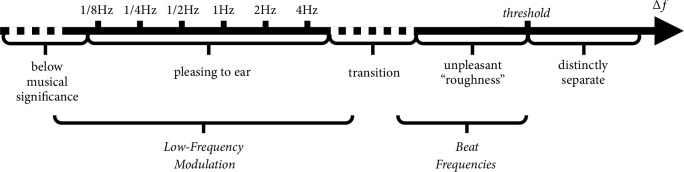
Types of interharmonic modulation on the scale of Δ*f*.

Interharmonic modulations with ∆*f* that increase towards a certain threshold are known to become increasingly dissonant, and, as coined by Helmholtz, are known as beating frequencies [8]. Interharmonic modulations with small ∆*f*, on the other hand, contribute to the harmonious effect perceived in consonance [65].  Figure 3 illustrates this.

### 3.2. Perceptual Responses across the** ∆***f*-$\overset{-}{f}$ Feature Space

**Figure 4:**
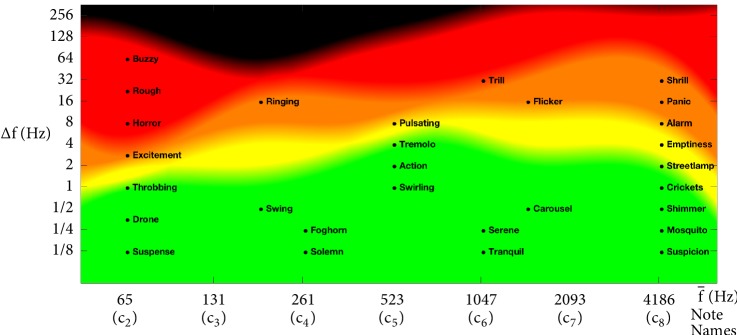
Example of auditory responses triggered by pure-tone frequencies on the horizontal axis modulated at frequencies on the vertical axis. Green, yellow, orange, red, and black indicate pleasing, somewhat pleasing, unpleasant, dissonant, and beyond beating range, respectively.

It is known that different combinations of notes contribute to different emotive valences [66]. This too may be decomposed into a sum of its harmonics. Hence, further to the consonances and dissonances, emotive responses may also be mapped onto the interharmonic plot. Although, as one might imagine, such responses would be different for every individual, we can plot the response for an individual as an example.  Figure 4 shows an example of auditory responses triggered in the mind of the (first) author when exposed to frequencies in the horizontal ($\overset{-}{f}$) axis modulated by frequencies in the vertical (**∆*f***) axis. The value of $\overset{-}{f}$ is indicated in the horizontal axis in both Hz and its corresponding note names. The degree of pleasure derived from interharmonic modulation is coded in the colored background as a reference. The green regions are perceived to be pleasing, yellow as somewhat pleasing, orange as unpleasant, but not to the point of annoying, red as dissonant, and black as beyond beating range. The black dots mark the locations of the thoughts or emotions labelled. This shows that interharmonic modulations bring about a large variety of thoughts or emotions. If several of these are triggered simultaneously when just one pair of notes sound simultaneously, one can imagine how ten fingers on a piano or all the instruments in an orchestra could combine several (thoughts or emotions) to paint stories on the interharmonic feature-space over time.

### 3.3. Intervals and Second-Order Modulations on the ∆*f*-$\overset{-}{f}$ Feature Space

**Figure 5:**
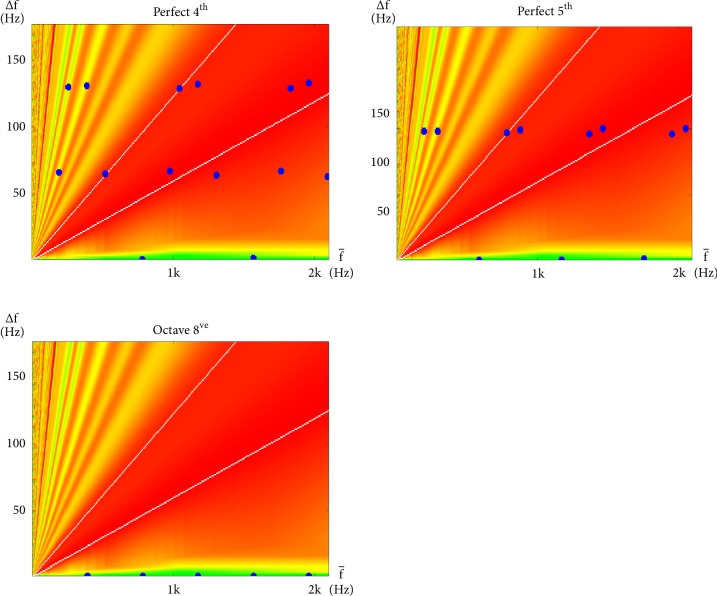
Interharmonic plots for all intervals within an octave regarded, in classical music theory, to be perfectly consonant with a root of g_3_. These are, namely, the Perfect 4^th^ (g_3_ and c_4_), Perfect 5^th^ (g_3_ and d_4_), and Octave (g_3_ and g_4_) intervals.

**Figure 6:**
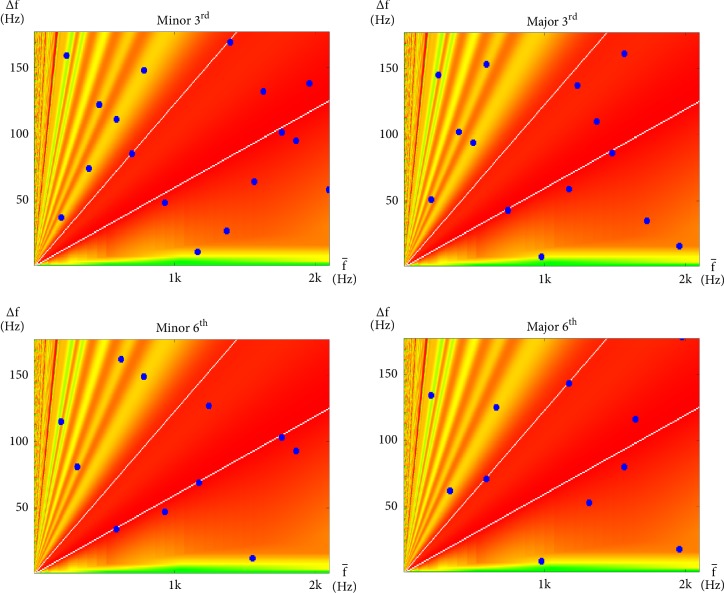
Interharmonic plots for all intervals within an octave regarded, in classical music theory, to be imperfectly consonant with a root of g_3_. These are, namely, the Minor 3^rd^ (g_3_ and b^b^_3_), Major 3^rd^ (g_3_ and b_3_), Minor 6^th^ (g_3_ and e^b^_4_), and Major 6^th^ (g_3_ and e_4_) intervals.

**Figure 7:**
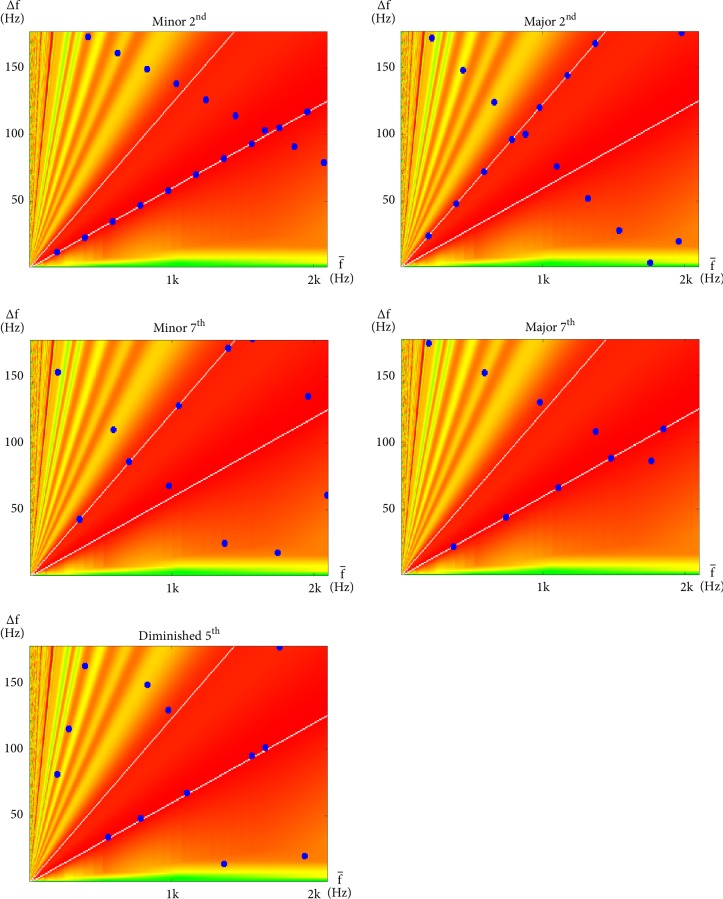
Interharmonic plots for all intervals within an octave regarded, in classical music theory, to be dissonant with a root of g_3_. These are, namely, the Minor 2^nd^ (g_3_ and a^b^_3_), Major 2^nd^ (g_3_ and a_3_), Minor 7^th^ (g_3_ and f_4_), Major 7^th^ (g_3_ and f^*♯*^_4_), and Diminished 5^th^ (g_3_ and d^b^_4_) intervals.

The interharmonic modulations of each interval within an octave are similarly plotted in Figures 5, 6, and 7. However, this time, the plots are in the linear scale. Green, yellow, orange, and red, again, represent regions of different degrees of consonance or dissonance according to the same color scheme as Figure 4. However, because this time both horizontal and vertical axes are in the linear scale, consonance-dissonance levels that populate the space on the nonlinear plot in Figure 4 now populate lower right regions of these linear plots. The remaining upper left regions are then populated with dissonance levels from [12]. These colors provide a simple background reference for the dark blue dots that each represent a modulation at their corresponding ∆*f* and $\overset{-}{f}$ values, which results from the summation of neighboring pairs of sinusoids (at frequencies $\overset{-}{f}+∆f/2$ and $\overset{-}{f}-∆f/2$) of the notes specified by the indicated interval. Also, for reference, are the two white lines that run across each plot, indicating the locations where the values of ∆*f* coincide with a semitone (gentler slope) and a tone (steeper slope) of the corresponding values of $\overset{-}{f}$ (where $∆f={(2}^{1/12}-1)\overset{-}{f}$ and $∆f={(2}^{2/12}-1)\overset{-}{f}$, resp.). The semitone and the tone are regarded as the most dissonant intervals up to halfway in either direction around the cyclic chroma [12, 21, 54].

The plots of perfect consonances are presented in Figure 5. These intervals are described with a bit of a dilemma in classical music theory [67]. They may be described as so consonant that they sound almost like one note. As such, their use contributes in a limited way to harmony [15]. For example, the use of perfect fifths is forbidden in parallel motion and octaves are regarded as the same note in a different register [42].

The interharmonic plot reveals the perceived traits of each category of intervals in a way that explains why they sound the way they do, and in a way music theory alone has never been able to. As shown in Figure 5, the constellations formed by interharmonic modulations of perfect intervals line up almost horizontally (While the methods used in this study are applicable with any form of tuning, only equitempered tuning is assumed in the computations in this section. This is consistent throughout this paper, unless otherwise stated.). Since each point that falls on the same horizontal has the same ∆*f*, this means that they modulate synchronously and may be perceived collectively as a single modulation. This may be interpreted as fewer modulating microevents taking place, making them less interesting than other consonance intervals.

Dissonant intervals are presented in Figure 7. As can be seen in the figure, these intervals have points that fall mostly within the central dissonant region and line up along the two dissonant lines. Evenly spaced points along a line that passes through the origin also reveal that their ∆*f* share a harmonic relationship. This has a similar (although this is somewhat lesser) redundant effect to that of the synchronous modulation described with perfect consonances.

Consonances that properly contribute to harmony are called imperfect consonances [67] and are presented in Figure 6. As can be seen in the figure, imperfectly consonant intervals have points better distributed. This may be interpreted as erratic modulations that create a continuous stream of unpredictable events to stimulate aural attention, and thus, interest.

A lot of work has already been done on interharmonics since Helmholtz [12, 19–21, 24, 25]. While the main focus of this work is not interharmonics, one purpose of this section is, nevertheless, to provide sufficient background to complete our theory of how the human experience of stationary harmony is based around modulations of both interharmonic and subharmonic nature. From the interharmonic plots in Figures 5–7, a simple predictor of dissonance may be identified to be

(10) $$\begin{array}{c} C\left(∆\hat{f}\right)=C\left(\frac{∆f}{\overset{-}{f}}\right)=\overset{n}{\underset{i=1}{\sum }}\left[2^{r_{lower}/12}-1<\frac{{∆f}_{i}}{{\overset{-}{f}}_{i}}<2^{r_{upper}/12}-1\right] \end{array}$$

where $∆\hat{f}$ will be our shorthand for $∆f/\overset{-}{f}$, $C(∆\hat{f})$, or $C\left(∆f/\overset{-}{f}\right)$ referring to the number of interharmonic modulations that fall within the central region of dissonance region, *i* iterates through all interharmonic modulations on the plot, *n* is the total number of modulations considered, ∆*f*_*i*_ and ${\overset{-}{f}}_{i}$ refer to the pair of ∆*f* and $\overset{-}{f}$ that describe the *i*th interharmonic modulation, respectively, and *r*_*lower*_ and *r*_*upper*_ define the lower and upper boundaries of the region on the interharmonic plot, respectively.

In this section, we have seen how interharmonic modulations are significant to our perception of consonance, dissonance, and emotive response in music. When listening to a duet of instruments with no overtones such as a sinewave theremin or a very pure musical saw, we realize that consonance, dissonance, and emotion remain present even in harmony without harmonics (i.e., across a well-spaced pair of fundamental frequencies alone). This is just one amongst the several different ways [12, 13, 17, 28, 68, 69] from which we can deduce that interharmonic modulations cannot be the only determinant of our perception of harmony, which thereby leads to our hypothesis on subharmonic modulations.

## 4. Subharmonic Modulations

Apart from the modulations that arise from the summation of adjacent harmonic sinusoids across differing notes, we can (as explained above) deduce that another category of modulations is significant to our perception of harmony. We call these subharmonic modulations. There are two levels of subharmonic modulations, which we dub subharmonic wave formation and subharmonic wave deformation. In this section, we will show how these are significant to our perception of not only stationary harmony, but also transitional harmony.

**Figure 8:**
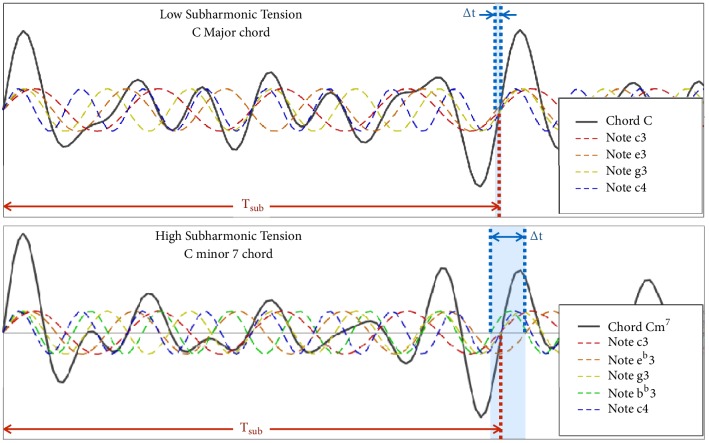
Subharmonic wave formation and deformation in the C and Cm^7^ chords.

Figure 8 shows the waveforms of a C Major chord (C) and a C minor 7 chord (Cm^7^) composed of the fundamental sinusoids of each composite note. We let each sinusoid start at phase zero since; for purpose of example, we are only interested in wave period. Only the fundament needs to be considered for the same reason. In both cases, the waveform resultant of this summation repeats at a frequency approximately subharmonic to all its composite waveforms. In the figure, its period is marked *T*_*sub*_. We call this subharmonic wave formation and say that *T*_*sub*_ is a common subharmonic to all its composite waveforms.

In the case of the C chord, as shown in the figure, each composite sinusoid crosses zero at nearly the same point around *t* = *T*_*sub*_. As marked in the figure, Δ*t* (which is the difference between the first and the last negative-to-positive zero-crossing around the *t* = *T*_*sub*_ region) is small. However, in the case of the Cm^7^ chord, Δ*t* is much larger. One can imagine that each successive period of the resultant waveform looks less and less like the first as it gets more and more deformed. This happens slowly for the C chord because of the small Δ*t* but faster for the Cm^7^ because of the large Δ*t*. We call this subharmonic wave deformation. Supplementary [Supplementary-material supplementary-material-1] compares subharmonic wave deformation in a low-tension C chord to that in a high tension Cm7 chord.

Recalling our wave equation from (3), we can rewrite *A*cos⁡*ω*_1_*t* + *B*cos⁡*ω*_2_*t*, or *A*cos⁡2*πf*_1_*t* + *B*cos⁡2*πf*_2_*t*, as

(11) $$\begin{array}{c} A\cos2\pi f_{1}t+B\cos2\pi f_{2}t=A\cos2\pi \left(k_{1}f_{sub}+\Delta f_{1}\right)t+B\cos2\pi \left(k_{2}f_{sub}+\Delta f_{2}\right)t \end{array}$$

where *f*_*sub*_ is an approximate common factor of *f*_1_ and *f*_2_, *k*_1_ and *k*_2_ are integer multipliers, and Δ*f*_1_ and Δ*f*_2_ are small values that balance the equation by making up for the discrepancies that arise with finding a common factor.

In (11), two fundamental frequencies *f*_1_ and *f*_2_ are described as the multiple of a lower subharmonic frequency that is common to them (*f*_*sub*_). We call this their* common subharmonic*.

Since all harmonics are multiples of their fundamental, a subharmonic to any fundamental would inherently be subharmonic to all its harmonics. For this reason, only the fundamental of each note needs to be considered.

Since harmony in music is commonly composed of more than just two notes, we generalize this to describe fundamentals and common subharmonics from any number of notes to get

(12) $$\begin{array}{c} \overset{N}{\underset{i=1}{\sum }}A_{i}\cos2\pi f_{i}t=A_{1}\cos2\pi \left(k_{1}f_{sub}+\Delta f_{1}\right)t+A_{2}\cos2\pi \left(k_{2}f_{sub}+\Delta f_{2}\right)t+\cdots +A_{i}\cos2\pi \left(k_{i}f_{sub}+\Delta f_{i}\right)t+\cdots +A_{N}\cos2\pi \left(k_{N}f_{sub}+\Delta f_{N}\right)t \end{array}$$

where *N* is the number of notes in the chord, *i* cycles through each of them, and *A*_*i*_ is the amplitude coefficient of note *i*.

Beyond this point, it would be easier to visualize subharmonics in the time domain. With the fundamental frequency of note *i* given by

(13) $$\begin{array}{c} f_{i}=k_{i}f_{sub}+\Delta f_{i} \end{array}$$

the fundamental period of each note *i* is then

(14) $$\begin{array}{c} t_{i}=\frac{1}{f_{i}}=\frac{1}{k_{i}f_{sub}+\Delta f_{i}} \end{array}$$

where *t*_*i*_ is the fundamental period of the note.

Hence, the period of any common subharmonic can be expressed as *k*_*i*_*t*_*i*_. We can then compensate for nonintegral discrepancies in period rather than in frequency. In doing so, we get

(15) $$\begin{array}{c} T_{sub}=\frac{1}{f_{sub}}=k_{i}t_{i}+\Delta t_{i} \end{array}$$

for all *i*, where *T*_*sub*_ is the common subharmonic wave period (we will simply say common subharmonic) of the chord. What carries over as *k*_*i*_*t*_*i*_ is essentially just the *k*th subharmonic of note *i* which lies in the region of *T*_*sub*_. Since this is true for all pairs of *k*_*i*_ and *t*_*i*_ across all values of *i* when they are each balanced by appropriate *t*_*i*_, *i* may be dropped from the left hand side of the equation.

Although the common subharmonic was introduced as the period between primary zero crossings as in Figure 8, we shall, for computational simplicity, redefine it as the mean of *k*_*i*_*t*_*i*_ across all notes of the chord. Hence,

(16) $$\begin{array}{c} T_{sub}=\overset{-}{k_{i}t_{i}} \end{array}$$

**Figure 9:**
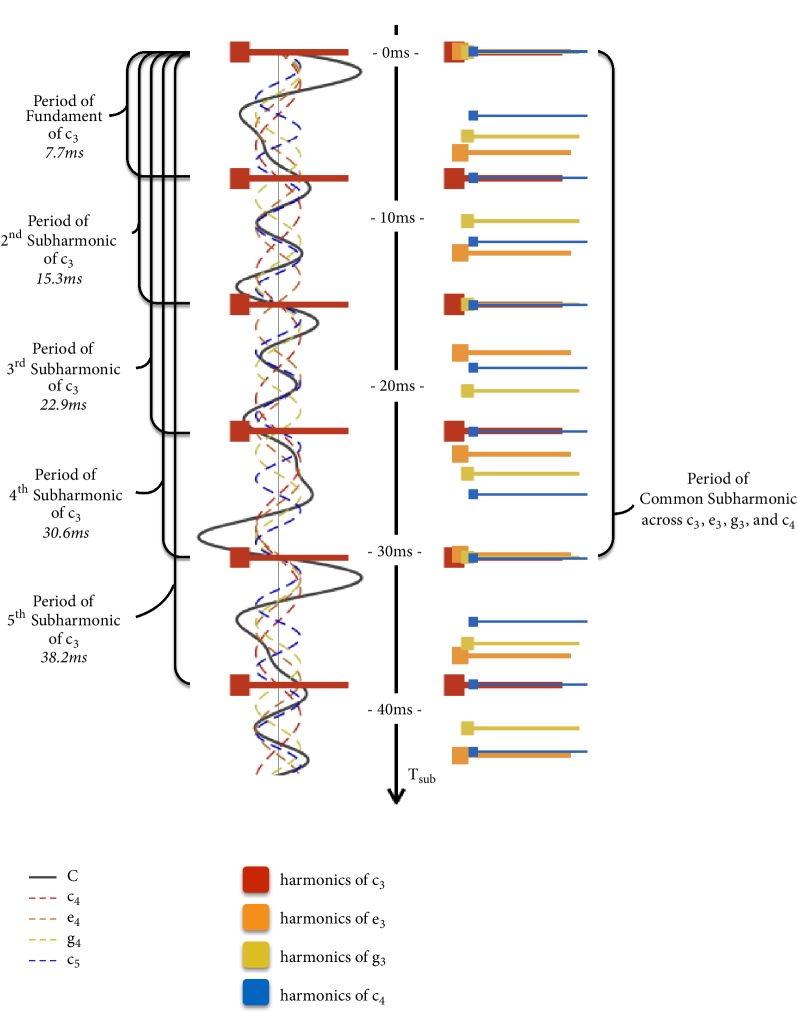
Plotting subharmonic wave periods of the C Major chord.

Figure 9 shows how the period of each subharmonic in the C Major chord from Figure 8 may be plotted. The left column first shows how the period of each subharmonic of c_3_ may be plotted in red. The right column then extends this to every remaining note in the chord, with orange, yellow, and blue for the notes e_3_, g_3_, and c_4_, respectively. It may be seen in the right column that a subharmonic period from every note in the chord nearly coincides at around 30 ms. Hence, we say that this is its common subharmonic, *T*_*sub*_, as defined in (16).

Having reduced the waveform plot to subharmonic periods in the vertical axis, we can represent time spanned by each subharmonic in the horizontal axis. We will do this for a song stanza in the next section, in a subharmonic plot.

### 4.1. Subharmonic Modulations in Stationary Harmony

**Figure 10:**
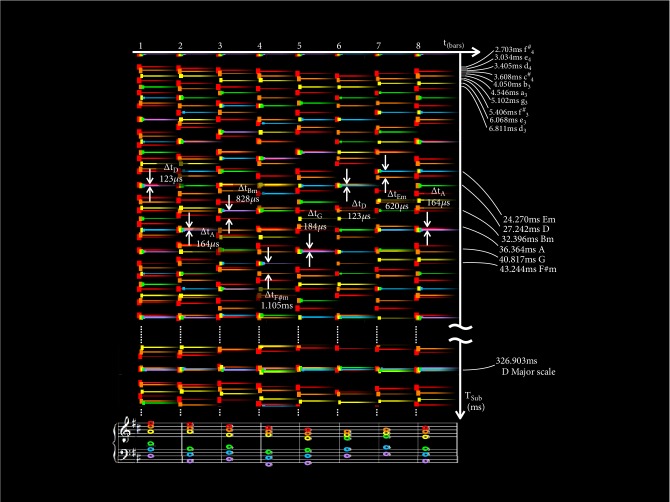
Subharmonic plot of the opening stanza of Pachelbel's Cannon in D with period in milliseconds on the vertical axis and time in bars on the horizontal axis. Subharmonics are colored to match the color of their corresponding notes on the music score below. The subharmonic tensions of each chord, ∆*t*, are marked out on the plot with white arrows. Significant wave periods, along with common subharmonic periods, *T*_*sub*_, are marked against the vertical axis on the right. In the interest of visiting all common chords of the key, Em is used in the 7^th^ bar instead of G, which already occurs in the 5^th^ bar. Considering the fact that this example is not used for transitional harmony, all chords are presented in its root position at the expense of introducing parallel 5^th^s in the interest of normalization for fairer comparison.

Figure 10 shows an example of a subharmonic plot. In the horizontal axis there is time in bars and in the vertical axis there is the subharmonic wave period in milliseconds. Note that the subharmonic axis runs top down to put shorter wave periods at the top because they correspond to higher frequencies. Larger wave periods, which correspond with lower frequencies sit conversely at the bottom. The tails that run horizontally represent the span of time covered by each note. Subharmonics are colored to match their corresponding notes on the music score. For example, in the first bar, all subharmonics of f^#^_5_ are marked out in red, followed by d_5_ in orange, a_4_ in yellow, d_4_ in green, a_3_ in blue, and d_3_ in purple. The musical score runs in parallel at the bottom of the plot as reference. Once again, all plots and computations in our examples assume equal temperament unless stated otherwise. This example shows the opening stanza of Pachelbel's Cannon in D [70] and focuses on stationary harmony, leaving transitional harmony to a later example.

*Subharmonics*. For every bar, the dashes that flush with the reference point at 0 ms mark 0 × *t*_0_. Carrying on top down with each bar in accordance to color, we get subharmonics at 1 × *t*_0_, 2 × *t*_0_, 3 × *t*_0_, 4 × *t*_0_, etc.

*Notes and Melody Line*. Since the topmost dash of each color for every bar below the 0 ms reference represents 1 × *t*_0_, they relate to the fundamental period of each note; of these, the topmost ones of every bar across all colors mark the melody line, f^#^_5_-e_5_-d_5_-c^#^_5_-b_4_-a_4_-b_4_-c^#^_5_. (They are red in this particular example.) Hence, it is easy to interpret the melody line in a subharmonic plot. The periods, *t*_*i*_, of each note of the melody are marked against the vertical axis in milliseconds as well as their common note names.

*Chords and Coincidence*. Common subharmonics may be visualized in regions with the (approximate) coincidence of dashes of every color. Again, the common subharmonics (*T*_*sub*_) of each chord in the stanza are marked out against the vertical axis in both milliseconds and their respective chord names.

*Key*. Every note of the diatonic shares a common subharmonic. Hence, it is possible to identify the key of a song by its common subharmonic, assuming minimal deviations from its key. The common subharmonic associated with the key of this song is marked out much further down the plot. Dotted lines indicate discontinuity. (This part of the figure is plotted in just intonation to avoid the snowballing of Δ*t*_*i*_ to better illustrate this.)

*Stationary Tension*. Most of the time, contributing subharmonics from different notes are not precisely coincident. Major chords have better coincidence than minor chords, and triads coincide better than sevenths and extended chords. With subharmonic modulations, perceptual tension arises with the noncoincidence of common subharmonics. Noncoincidence is measured by an overall Δ*t* as reflected in Figures 8 and 10. We call this its (stationary) subharmonic tension.

This Δ*t* is given by the difference between the largest and smallest subharmonics in the chord that coincides around *T*_*sub*_.

(17) $$\begin{array}{c} \Delta t={\left[k_{i}t_{i}\right]}_{max}-{\left[k_{i}t_{i}\right]}_{min} \end{array}$$

where [*k*_*i*_*t*_*i*_]_*max*_ and [*k*_*i*_*t*_*i*_]_*min*_ denote the largest and the smallest subharmonics in the chord that (nearly) coincides around *T*_*sub*_ (mathematically, they are the maximum and minimum values of *k*_*i*_*t*_*i*_, resp.).

Δ*t* and *T*_*sub*_ are the primary features of stationary tension. Δ*t* may be normalized by expressing it like a duty cycle by taking

(18) $$\begin{array}{c} ∆\hat{t}=\frac{\Delta t}{T_{sub}} \end{array}$$

From Figure 3 in the section on interharmonic modulation, recall that dissonances increased and decreased with interharmonic modulation frequency while consonances behaved inversely. This happens only within a certain range. When interharmonic modulation frequency shrinks to the brink of zero, it falls below musical significance.

**Figure 11:**
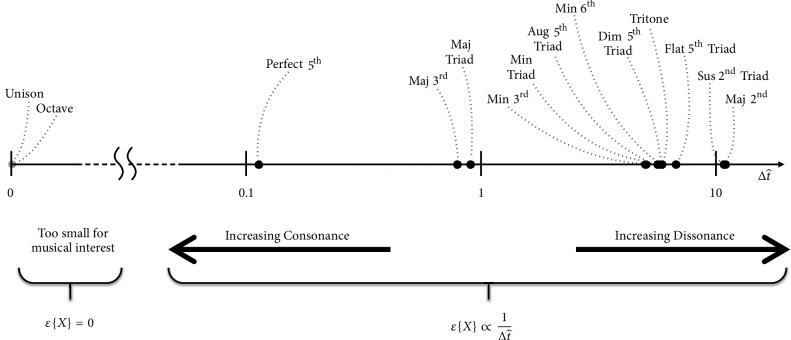
The effect of harmony, *ε*{*X*}, on the scale of subharmonic tension, $∆\hat{t}$.

Subharmonic tension behaves similarly.  Figure 11 describes different types of harmony on the subharmonic tension scale. As can be seen in the figure, our response to subharmonic tension is likewise. Perceived dissonances increase and decrease with subharmonic tension while perceived consonances behave inversely within common range. Mathematically,

(19) $$\begin{array}{c} \varepsilon \left\{X\right\}\propto \frac{1}{{\Delta \hat{t}}_{X}} \end{array}$$

where *ε*{X} is the* harmonious effect of chord X* and ${\Delta \hat{t}}_{X}$ is its stationary subharmonic tension (its $\Delta \hat{t}$).

However, as described in the figure, modulations from subharmonic tension fall below musical significance; the effect of harmony drops to zero as modulations from subharmonic tension fall below musical significance. Hence, where ${∆\hat{t}}_{threshold}$ is the said threshold of musical significance, as $∆\hat{t}<{∆\hat{t}}_{threshold}$,

(20) $$\begin{array}{c} \underset{∆\hat{t}\to 0}{\lim}\varepsilon \left\{X\right\}=0 \end{array}$$

Thus, perceptual tensions and consonances are experienced in slew-like modulations of the waveform at common subharmonic locations. (This is the effect of periodically changing phase relationships amongst the contributing waveforms, for which Δ*t* is a measure.) While there may be several common subharmonics for every chord within reasonable range, we theorize that our ears identify most with the shortest few. Subharmonic consonances are described by gentler modulations (small Δ*t*) at the shortest common subharmonic locations (short *T*_*sub*_), while subharmonic dissonances are described by more turbulent ones (associated with absence of small Δ*t* at short *T*_*sub*_).

The sensation of a chord can be highly complex, with different tensions and consonances perceived simultaneously, an experience inadequately represented by a single term for dissonance. Attempting to rate every chord by its dissonance level alone can be compared to rating every variety of chocolate in a candy store by only how sweet or bitter it is. The advantage of ∆*t*, as opposed to existing correlates of harmony [3, 13, 43, 54], is the way it explains abstract notions of perceptual tensions and consonances by ascribing them to regions across the subharmonic spectrum with a strong sense of attribution or identification. While, for purpose of illustration, Figures 9 and 10 have shown examples where a modal *T*_*sub*_ (shortest *T*_*sub*_ with smallest ∆*t*) is easiest to identify, we theorize complex chords with ambiguous *T*_*sub*_ (where it is difficult to attribute the collection of modulations experienced to a single modal); our ears often identify with several common subharmonics simultaneously. In other words indeterminate cases could possibly arise with particularly discordant harmonies without small ∆*t* at short *T*_*sub*_. Thus, for programmatic analysis of a large number of chords, it is, nevertheless, useful to have a single term to represent the overall dissonance of each chord. For this, we use

(21) $$\begin{array}{c} \tilde{∆t}={n\left(\frac{1}{\sum_{n:m}^{}\left(1/{T_{sub,j}\left({∆t}_{j}\right)}^{c}\right)}\right)}^{1/c} \end{array}$$

where a single term, $\tilde{∆t}$, represents the overall subharmonic tension, *T*_*sub*,*j*_ and ∆*t*_*j*_ refer to individual candidates of *T*_*sub*_ and ∆*t* with *j* iterating through each candidate pair, *c* is the preemphasis (while 1/*c* serves as “post de-emphasis”), and Σ_*n*:*m*_ denotes summing over the *n* smallest values out of a range of *m* values considered. In our work, *n* is always chosen to be half of *m* unless stated otherwise. Note that *T*_*sub*,*j*_ here serves as a weighting factor to weight down higher subharmonics, which, as aforementioned, are less significant. Inverting before (and rectifying after) summation mimics our hearing by allowing smaller values of ∆*t*_*j*_ to contribute better towards a smaller $\tilde{∆t}$.

We will see how representative $\tilde{∆t}$ is of stationary harmony in the next section. But before that, we will first explain subharmonic modulations in transitional harmony.

### 4.2. Subharmonic Modulations in Transitional Harmony

While stationary harmony studies chord sonorities (how a chord sounds on its own), transitional harmony deals with chord progressions and resolutions (how chords transit from one to another). It is remarkable how a low tension (consonant) chord can transit to a high tension (dissonant) one yet still bring about the perceptual effect of tension release (resolution) [18]. From this it may be deduced that transitional harmony stands largely independent of stationary harmony, even though both are considered when assigning harmony in composition. Even though numerous studies have been conducted on stationary harmony from the psychoacoustic approach, work on transitional harmony remains primarily nonpsychophysical.

**Figure 12:**
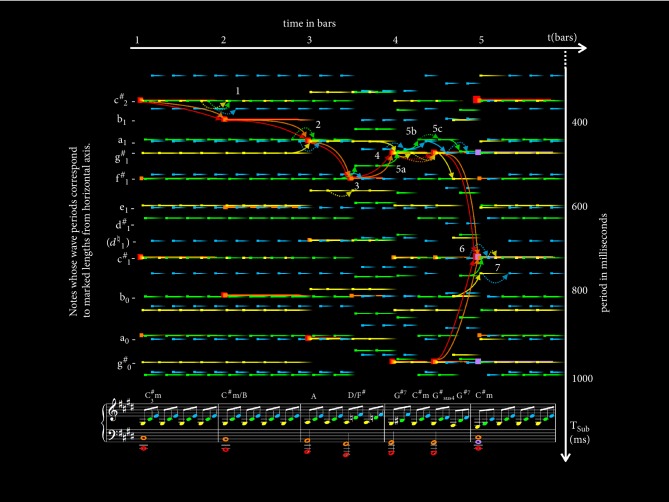
Subharmonic plot of the opening line of Beethoven's Moonlight Sonata with period in milliseconds on the vertical axis and time in bars in the horizontal axis. Subharmonics are colored to match their corresponding notes on the music score. Names of relevant notes are marked out on the left, at *T*_*sub*_ values corresponding to their wave period. The region of each transition is numbered in white. Colored arrows follow voice leading along the notes across chord changes.

Traditional classical music theory uses the term resolution to describe the perception of tension released when a chord is suitably followed by another chord [18]. With subharmonic modulation, we theorize that these abstract perceptions of tensions released may be identified and quantified in the perceived trajectories of subharmonics as one chord progresses to the next.  Figure 12 illustrates this.

Figure 12 shows the opening line of Beethoven's Moonlight Sonata [71]. Before we begin our analysis, one should note that unlike Pachelbel's Cannon the use of* arpeggios* (broken chords) means that notes contributing to the harmony may not necessarily start at the same time, but, when the sustain pedal on the piano is applied, they sustain and overlap until the end of each bar. The names of the chords formed by the notes are labelled along the top of the score to aid the reader in this analysis. Another thing to note would be the fact that this piece maintains a strong sense of* voice leading *[72], which means that each note from a chord has strong progressive associations with a note from the previous and another from the succeeding chord. The subharmonics of all notes that are associated in this way (i.e., of the same voicing) across the song are coded with the same color to aid the reader in this analysis. For example, all notes in red on the music score represent the bass (lowest) notes throughout the song, and every subharmonic of these notes is portrayed in red.

We theorize that in chord transitions every subharmonic (*k*_*i*_*t*_*i*_) that (nearly) coincides around the common subharmonic (*T*_*sub*_) of a succeeding chord is perceived to transit from the nearest corresponding (i.e., of the same voicing) subharmonics in the preceding chord. These transitions are marked out by the arrows in Figure 12, which are colored according to the notes they are associated with. Arrows are usually convergent (with the exception of, for example, a basic triad progressing onto an extended chord of the same root) because the subharmonics of the succeeding chord always identify with a common subharmonic whereas those of the preceding chord usually do not.

The central hypothesis of transitional subharmonic theory is that perceptual tension resolution, which is so often described in traditional music theory but never physically identified in acoustics, lies in the degree of convergence seen here.

Assuming transition to be abrupt (since notes do not commonly glide from one pitch to another in music) we compute a Δt for the succeeding common subharmonic and a Δt for its preceding corresponding subharmonics and simply measure this degree of convergence as the difference between the two. As such,

(22) $$\begin{array}{c} ∆∆t={∆t}_{p}-{∆t}_{s} \end{array}$$

where ∆*t*_*s*_ refers to the ∆*t* of the succeeding chord and ∆*t*_*p*_ refers to the ∆*t* defined by its nearest preceding subharmonics.

This can be normalized by dividing by *T*_*sub*_ such that

(23) $$\begin{array}{c} ∆∆\hat{t}=\frac{{∆t}_{p}-{∆t}_{s}}{T_{sub}} \end{array}$$

where $∆∆\hat{t}$ denotes normalized ∆∆*t* and *T*_*sub*_ refers to that of its succeeding chord.

∆∆*t* is, thus, a quantification of the tension; Δ*t* is released over the transition at the wave period of the succeeding common subharmonic.

According to our theory, tension resolution is perceived in the release of this tension across each transition. Thus, mathematically,

(24) $$\begin{array}{c} \varepsilon \left\{X_{1}\to X_{2}\right\}\propto {∆∆\hat{t}}_{X_{1}\to X_{2}} \end{array}$$

where *ε* denotes the perceptual resolving effect of tension release and ${∆∆\hat{t}}_{X_{1}\to X_{2}}$ denotes the $∆∆\hat{t}$ across the transition of chord X_1_ to chord X_2_.

Since resolution (tension release) [18, 42] in harmony progression is perceived in the convergence of $∆\hat{t}$, what we will refer to as* complication* (build-up of tension or negative resolution) is seen in its divergence, where $∆∆\hat{t}<0$ and *ε*{X_1_ → X_2_} is negative.

**Figure 13:**
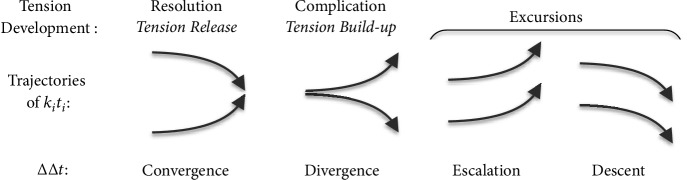
Trajectories of *k*_*i*_*t*_*i*_ in different states of tension development (states of convergence).

Three possibilities arise when looking at *T*_*sub*_ and ∆*t* from this perspective, by which we can divide transitional harmony into three classes. As illustrated in Figure 13, these are as follows.

(1) Resolution, also called tension release: this is the most common occurrence and occurs with the* convergence* of Δt (i.e., ∆*t*_*p*_ > ∆*t*_*s*_) and a positive ∆∆*t*. The larger the ∆∆*t*, the larger the perceptual tension release.
(2) Complication, also called tension buildup: this is the least common occurrence and occurs with the* divergence* of Δ*t* (i.e., ∆*t*_*p*_ < ∆*t*_*s*_) and a negative ∆∆*t*.* Just as negative aesthetics may be used expressively in a painting, it may similarly be used in music* [73]. The larger the magnitude of∆∆*t*, the larger the perceptual tension buildup. Complications usually only occur when the preceding *T*_*sub*_ is equal or nearly equal to the succeeding *T*_*sub*_. Musically speaking, it usually occurs when a simpler chord is followed by a more complex chord of the same root.
(3) Excursion*:* Because of the circular nature of the musical chroma, the preceding *T*_*sub*_ and the succeeding *T*_*sub*_ may be computed to differ by up to 6 semitones in either direction. When the difference is 1 or 2 semitones, this corresponds to a neighboring note, and the collective (uplifting or detrimental) effect of melodic movement (i.e., melody) across each note of the chord can overpower the effect of harmony. In such cases, our ears are persuaded to identify ∆*t*_*p*_ with [*k*_*i*_*t*_*i*_]_*max*_ − [*k*_*i*_*t*_*i*_]_*min*_ of the nearest preceding *T*_*sub*_. When this happens, [*k*_*i*_*t*_*i*_]_*max*_ and [*k*_*i*_*t*_*i*_]_*min*_ move in the same direction; hence,* neither convergence nor divergence is perceived. *There are 2 such cases as follows.
  - Escalation: this occurs when each [*k*_*i*_*t*_*i*_] shortens simultaneously, *T*_*sub*_ shortens by a factor equivalent to 1 or 2 semitones (2^1/12^ to 2^2/12^ times), and *f*_*sub*_ rises, producing the uplifting effect of melodies rising by 1 or 2 semitones.
  - Descent*:* this occurs when each [*k*_*i*_*t*_*i*_] lengthens simultaneously, *T*_*sub*_ lengthens by a factor equivalent to 1 or 2 semitones (2^1/12^ to 2^2/12^ times), and *f*_*sub*_ falls, producing the detrimental effect of melodies falling by 1 or 2 semitones.

It is fascinating to note how the perceptual development (build-up and resolution) of tension that is so often described in music [18, 42] but never identifiable with an acoustic attribute may here be visualized in the convergence and divergence of common subharmonics. Figure 13 further illustrates how *k*_*i*_*t*_*i*_ trajectories reflect the development of tension build-up and release. Additionally, trajectories for excursions are illustrated in the same figure.

Returning to Figure 12, the transitions between each chord are labeled 1 to 7 in the figure and correspond to 1 to 7 as follows.

(1) The song starts off with a C^#^m chord. Hence, the common subharmonic is observed around a wave period of c^#^. Our ears adhere especially to the shortest one, which is at c^#^_2_. Large Δt is attributed to the complex tensions within a minor chord. At the region marked 1, this transits to a C^#^m/B chord. The tension built up with the divergence of Δt may be visualized in the divergence of the arrows in the figure (of which the dotted ones across the plot are used to indicate the continuation of subharmonics, i.e., *k*_*i*_*t*_*i*_that do not change). Both perceptually in music and acoustically, as defined above, this translates to a further complication to the existing minor tension.
(2) At region 2, there is a convergence to a momentary (half-bar) low-tension A chord. The uplifting effect of a large tension release, $∆∆\hat{t}\gg 0$, is counterbalanced by the detrimental effect of a falling melodic sequence (lengthening *T*_*sub*_), adding to the complexity of the song.
(3) At region 3, A transits to a D/F^#^, which is a Neapolitan chord. The low f^#^ bass extends over 2 octaves below the treble notes, putting a strong *T*_*sub*_ at a nonroot period of f^#^_1_ and creating an amount of stationary tension that is unusual for a major chord. (In such cases, there is usually another common subharmonic with lower Δ*t* but at a wave period corresponding to a root at a much larger *T*_*sub*_.)
(4) At region 4, the Neapolitan chord resolves to the Dominant 7^th^, marked G^#7^ in the figure, with a large perceptual resolution that is signature to  ^b^II^6^-V^7^ transitions in music [42]. This large tension release is visualized as a large convergence in the subharmonic plot as indicated by the arrows.
(5) Musically, the Dominant 7^th^ typically plays the role of building an anticipation for the upcoming return to the Tonic [42]. Beethoven enhanced this function particularly well with a double suspension with staggered resolutions in regions 5a through 5c. The subharmonic plot gives tangibility to the perceptual details with suspension-resolution long theorized about in music that can now be affirmed with visualization.
  - At region 5a, the transition from the G^#7^ progresses to what is labeled C^#^m. However, this C^#^m is functionally still a G^#^ with a double suspension of the 3^rd^ (b^#^) to a 4^th^ (c^#^) and the 5^th^ (d^#^) to a 6^th^ (e), respectively. The perceptual complication that arises with this transition can be visualized in the subharmonic plot as indicated by the divergence of the green and cyan arrows, respectively. The deviation of the suspended notes from the primary triad is visualized as a deviation of their *k*_*i*_*t*_*i*_ from *T*_*sub*_.
  - At region 5b, the tension resolution with the 6^th^ being resolved back down to the 5^th^ can be visualized in the subharmonic plot by its *k*_*i*_*t*_*i*_ resolving back to *T*_*sub*_ as indicated by the convergent cyan arrow. The continuation of the suspended 4^th^ is visualized in the dotted green arrow.
  - At region 5c, the tension resolution with the 4^th^ being resolved back down to the 3^rd^ can be visualized in the subharmonic plot by its *k*_*i*_*t*_*i*_ resolving back to *T*_*sub*_ as indicated by the solid green arrow. In preparation for a major resolution back to the upcoming tonic, Beethoven's touch of genius combines this resolution with a simultaneous complication in the introduction of the 7^th^ at this point. This is visualized in the deviation of its *k*_*i*_*t*_*i*_ away from *T*_*sub*_ as indicated by the divergent solid yellow arrow.
(6) At region 6, the Dominant 7^th^ is resolved back to the Tonic with a tension release unique to V_7_-tonic cadences that is so immense that it is has been long established as the de facto cadence for the end of musical passages [42]. This immense perceptual release of tension, too, is identifiable in the subharmonic plot. From the figure, it may be seen that the common subharmonic, *T*_*sub*_, of C^#^m (located at the period of c^#^_1_ this time, because of the g^#^_2_ in purple) lies right in the middle of two common subharmonics of G^#7^ (located at the periods g^#^_1_ and g^#^_0_). This unique subharmonic behavior allows our ears to quite possibly identify with both *k*_*i*_*t*_*i*_ for the preceding ∆*t* making ${∆\hat{t}}_{p}$ significantly larger than its ${∆\hat{t}}_{s}$. Its staggering convergence produces an immense sense of tension resolution with this transition.
(7) A final landmark that is interesting to note is at region 7, where the triad in the treble flips from the 1^st^ inversion to the 2^nd^ inversion while the chord remains unchanged. Notice that this brings about no change to both *T*_*sub*_ and $∆\hat{t}$ while $∆∆\hat{t}=0$. This, again, shows how subharmonic analysis agrees with music theory where, despite the change of notes, harmony remains the same at this point.

In this section, we have seen how, even in the context of transitional harmony, perceptual tensions and resolutions in a song may be visualized in its subharmonic modulation. We will move on to see how well numerical values computed with such modulations verify against listening tests and chord use statistics.

## 5. Experiment and Results

For both stationary and transitional harmony, tensions computed from our models show strong correlations with consonance rankings and historical chord use statistics.  Table 1 tabulates a summary of the results of our experiment.

We will explain each of these results in detail in the following subsections.

### 5.1. Stationary Harmony

For stationary harmony, we take the overall tension of a chord to be a simple weighted sum of *T*_∆*f*_ and *T*_∆*t*_

(25) $$\begin{array}{c} T_{∆f\;|\;∆t}=w_{i}T_{∆f}+w_{s}T_{∆t} \end{array}$$

where *T*_∆*f*∣∆*t*_ is overall tension, *T*_∆*f*_ and *T*_∆*t*_ are taken to represent the tensions contributed by interharmonic and subharmonic modulations, respectively (normalized by linearly scaling to fit between 0 and 1), and *w*_*i*_ and *w*_*s*_ are their weights, or summing coefficients respectively, where *w*_*i*_ + *w*_*s*_ = 1 and 0.61 and 0.39 are found to provide a good distribution.

We use a simple estimate of *T*_∆*f*_, taking

(26) $$\begin{array}{c} T_{∆f}=C_{1}\left(∆\hat{f}\right)+C_{2}\left(∆\hat{f}\right) \end{array}$$

where $C_{1}(∆\hat{f})$ and $C_{2}(∆\hat{f})$ are a tally of interharmonic modulations (given by (10)). By visual inspection of the interharmonic plot, regions of dissonance are defined by *r*_*lower*_ = 0.95 and *r*_*upper*_ = 1.1 for $C_{1}(∆\hat{f})$ and *r*_*lower*_ = 1.5 and *r*_*upper*_ = 2.8 for $C_{1}(∆\hat{f})$.

For *T*_∆*t*_, we use ${\left(\tilde{∆t}\right)}^{2}$, where $\tilde{∆t}$ is given by (21) preemphasized with *c* = 2.1 across a range of *m* = 5. (A preemphasis of just over 2 provided the sufficient discrimination without driving data into saturation. A broad range of *m*-values are suitable but we settled on a smaller value of 5 for computational simplicity.)

Numerous previous authors have performed notable work for stationary harmony both within and outside the psychophysical context [8, 12, 13, 18, 21–25, 43, 53, 62–64]. For dyads (intervals, or two-note chords) and triads (three-note chords), we the use precollated information in Tables 2–5 from Stolzenburg [43] for comparison. Dyads (intervals) are compared against the results of an average across 7 notable studies collated by Schwartz et al. [54] on a ranking of 12 chords. Stolzenburg adds the unison to Schwartz's list, which he reasonably assumes to be the most consonant, hence, we have appropriately included it as well. Triads are compared to results from an experiment by Johnson-Laird, Kang, and Leong [13] as cited in Stolzenburg [43]. For consistency with Stolzenburg's statistics in the comparison, these were first converted to ordinal rankings before computing the correlation as practised by Stolzenburg [43].  Table 2 lists our correlations for dyads and triads in stationary harmony against known relevant work as taken from Stolzenburg's [43]. A detailed tabulation of all available values for each chord is provided in the appendix.

### 5.2. Transitional Harmony

For transitional harmony, ∆∆*t* from (22) is suitable for hand-computation of transitional harmony across individual locations of succeeding common subharmonics, ∆*t*_*s*_, across the soundscape. While this is advantageous for visualizing individual complications and resolutions at multiple locations across the tensional soundscape, it requires manual identification of a modal ∆*t*_*s*_ for every transition which can be ambiguous for particularly discordant harmonies. For a consistent programmatic approach with larger datasets, we take the measure of* overall *∆∆*t* of a transition defined by

(27) $$\begin{array}{c} {\tilde{∆∆t}=\left(\frac{1}{n}\sum_{j=1,\;\;\forall {∆t}_{s,j}<\left(1/2\right)∆T_{sub}}^{N}{\left(\frac{∆∆{\hat{t}}_{j}}{{∆t}_{s,j}T_{sub,j}}\right)}^{c}\right)}^{1/c} \end{array}$$

where $\tilde{∆∆t}$ is representative of overall tension resolved, $∆∆{\hat{t}}_{j}$, ∆*t*_*s*,*j*_, and *T*_*sub*,*j*_ refer to individual candidates of $∆∆\hat{t}$, ∆*t*_*s*_, and *T*_*sub*_, respectively, *N* is the range of nodes considered, *j* iterates through all relevant common subharmonics of the succeeding chord, ∆*T*_*sub*_ denotes the distance between two adjacent *T*_*sub*,*j*_, Σ_*j*=1,  ∀∆*t*_*s*,*j*_<(1/2)∆*T*_*sub*__^*N*^ denotes summing across all values of 1 < *j* < *N* wherever ∆*t*_*s*,*j*_ is less than half the distance between the adjacent *T*_*sub*,*j*_ on either side, *n* is the number of nodes summed, and *c* is the preemphasis as explained with (21).

This effectively computes the preemphasized, weighted, and compensated mean ∆∆*t* across all eligible common subharmonics within a range of *N* for a given transition. *T*_*sub*_ weights down larger subharmonics which are less significant according to the theory. (It is a reciprocal as opposed to (21) because greater pleasure is associated with larger tension released.) ∆*t*_*s*,*j*_ compensates for the fact that, apart from tension resolution alone, stationary consonance also affects one's preference for the succeeding chord. ∆*t*_*s*,*j*_ < (1/2)∆*T*_*sub*,*j*_ effectively sets the criterion for a node to be considered a common subharmonic. In our experiments, we set *N* = 9. (A broad range of *N* will work, but we choose a smaller value for computational simplicity. Larger values may be required with larger range or dataset size.) In consideration of divergent transitions in the dataset, we set *c* = 1 (no preemphasis) because divergent transitions have negative ∆∆*t* which can be distorted by preemphasis.

With transitional harmony, conducting an accurate listening test is less straightforward. Rather than attempting to acquire a small number of fresh unproven opinions, it is reasonable to use statistics from a large number of well-esteemed premade decisions. A simple way to measure how well numerical values of subharmonic transition agree with the music theorists' school is to compare them with statistics of an expert music theorist's chord use. Capturing chord-use statistics from music score is again, however, a labor-intensive process requiring domain expertise [46, 47, 74]. Details such as melody-harmony discrimination, transition onset, and root ambiguity (e.g., Dm^7^/F versus F^6^) are often not precisely defined in a song. We find the largest relevant data readily available that also meets chord-spelling precision requirements in Tymoczko's* Study on the Origins of Harmonic Tonality* [45]. In this study, Tymoczko interpreted and recorded the statistics of 11,000 chord transitions from Palestrina's [75] corpus. Palestrina was highly regarded for his style of harmony by Helmholtz himself [76]. He is widely considered amongst music theorists to be the pinnacle of contrapuntal harmony [77].

Table 3 lists $\tilde{∆∆t}$ against frequencies of occurrence for each of the 17 most frequently used chords that follow V as read-off Tymoczko [45]'s chord tendency histogram. C, D, X↑, and X↓ indicate the convergence type of the progression. Just intonation was used as being opposed to equal temperament in this case to be consistent with Palestrina.

Their correlations are listed in Table 4. $\tilde{∆∆t}$ shows a significantly strong positive correlation of 0.903 with Palestrina's chord tendencies in general. It is close to perfect at 0.996 for resolutions since the programmatic version of the model was designed with resolutions in mind. Complications may be interpreted as the negative release of tension. Even though a large number of contributing $∆∆{\hat{t}}_{j}$ are negative, only one negative $\tilde{∆∆t}$ can be seen in the table due to the influence of nonnegative candidates. Nevertheless, $\tilde{∆∆t}$ shows a strong negative correlation of -0.761 with [45] for complications (agreeing with the fact that this resolution is negative). As earlier explained, with excursions the perception of a succeeding chord is also influenced by the rising or falling of parallel melodies. Unfortunately, descending excursions were insufficiently popular in Palestrina and only V-IV was being tallied. For escalating excursions, however, we have enough statistics to compute a correlation of 0.863. We have also computed the correlation across all other chords separately from complications (because, as explained, they correlate negatively) to be 0.970.

## 6. Discussion

*Addressing the Fundamental Questions of Psychoacoustic Harmony*. At this point, let us address the fundamental questions of psychoacoustic harmony as promised at the start of this paper in the context of subharmonic modulations. We will begin with question 2 and leave the first question for the last.

(2) We discussed the definition and explanation of stationary harmony, i.e.,* what sounds good* and* why*, or, mathematically, to quantify *ε*{*X*_*n*_}, where *ε*{} denotes* the harmonious effect of *and *X*_*n*_ represents chord *n*.
*With large subharmonic tension being perceived as dissonance while small subharmonic modulations are perceived as consonance, the aesthetics of a chord may be visualized in the subharmonic tension acting on its shortest common subharmonics. Mathematically, they are inversely related. As described by (19), *$\varepsilon \left\{X\right\}\propto 1/∆\hat{t}$.
(3) We have the definition and explanation of transitional harmony, i.e.,* what sounds good*,* why*, and* when*, or, mathematically, to quantify *ε*{*X*_1_ → *X*_2_}, where ‘→' denotes transition from one chord to another.
*The aesthetics of a chord transition may be visualized in the release of subharmonic tension at the shortest common subharmonics of the succeeding chord. As explained in (22) and indicated by the arrows in Figure 12, this refers to the transition to the shortest common subharmonics of the succeeding chord from the nearest subharmonics of the preceding chord. Thus, resolution (tension release) in a chord transition is perceived in the convergence of *$∆\hat{t}$* (where *$∆∆\hat{t}>0$*) while what we call complication (build-up of tension or negative resolution) is seen in its divergence (where *$∆∆\hat{t}<0$*). Mathematically, as described by (24), *$\varepsilon \left\{X_{1}\to X_{2}\right\}\propto {∆∆\hat{t}}_{X_{1}\to X_{2}}$.
(4) We have the following phenomena.
  - A chord that sounds better than another out of context can sound worse than being in context [42]. Given *ε*{*X*_2_} > *ε*{*X*_3_} this shows that *ε*{*X*_1_ → *X*_2_} < *ε*{*X*_1_ → *X*_3_}
*The section on subharmonic modulations differentiates between stationary tension and transitional tension. The tension release brought about by a transition to a chord may be large even for high tension succeeding chords. To prove this, we will use an example with E*^7^, *G, and Am*^7^*. Taking E*^7^ = {*b*_3_, *d*_4_, *e*_4_, *g*^#^_4_}, *G* = {*g*_3_, *b*_3_, *d*_4_, *g*_4_}*, and Am*^7^ = {*a*_3_, *c*_4_, *e*_4_, *g*_4_, *a*_4_}*, the stationary subharmonic tension for G and Am*^7^* may be computed by (18) to be *${∆\hat{t}}_{G}=0.902\%$* and *${∆\hat{t}}_{{Am}^{7}}=6.849\%$*, respectively. Thus, ε*{*G*} > *ε*{*Am*^7^}*, whereas the transitional subharmonic resolution (tension resolution) for E*^7^ → *G and E*^7^ → *Am*^7^* may be computed by (22) to be *${∆∆\hat{t}}_{E^{7}\to G}=8.783\%$* and *${∆∆\hat{t}}_{E^{7}\to {Am}^{7}}=10.748\%$*, respectively. Thus, ε*{*E*^7^ → *G*} < *ε*{*E*^7^ → *Am*^7^}* despite the fact that ε*{*G*} > *ε*{*Am*^7^}.
  (b) A chord that sounds better than another in one context can sound worse than being in another context [42]. Given *ε*{*X*_4_ → *X*_2_} > *ε*{*X*_4_ → *X*_3_} this shows that *ε*{*X*_1_ → *X*_2_} < *ε*{*X*_1_ → *X*_3_}
*With reference to (22) and our answer in question 3, since our ears identify the subharmonics of preceding notes that correspond to the succeeding common subharmonic, transitional harmony is contextual. Continuing from our answer to question 4a, we take D*^7^* to be D*^7^ = {*c*_4_, *d*_4_, *f*^#^_4_, *a*_4_}*. The transitional subharmonic resolution (tension resolution) for D*^7^ → *G and D*^7^ → *Am*^7^* may be computed by (22) to be *${∆∆\hat{t}}_{D^{7}\to G}=11.421\%$* and *${∆∆\hat{t}}_{D^{7}\to {Am}^{7}}=4.540\%$*, respectively. Thus, ε*{*D*^7^ → *G*} > *ε*{*D*^7^ → *Am*^7^}* despite the fact that ε*{*E*^7^ → *G*} < *ε*{*E*^7^ → *Am*^7^}.
(5) phenomenon that the transition from a low-tension chord to a high-tension one can still bring about the effect of tension release (resolution). Given *ε*{*X*_1_} < *ε*{*X*_2_} this shows that *ε*{*X*_1_ → *X*_2_} > 0.
*The answer to this is in the independence of stationary and transitional tension, as established in our answer to Question 4a*.
*Taking E* = {*b*_3_, *e*_4_, *g*^#^_4_}* and Am*^7^ = {*a*_3_, *c*_4_, *e*_4_, *g*_4_, *a*_4_}*, the transitional subharmonic resolution (tension resolution) for E* → *Am*^7^* may be computed by (22) to be *${∆∆\hat{t}}_{E\to {Am}^{7}}=4.323\%$*. The stationary subharmonic tension for E and Am*^7^* may be computed by (18) to be *${∆\hat{t}}_{E}=0.902\%$* and *${∆\hat{t}}_{{Am}^{7}}=6.849\%$*, respectively. Hence, ε*{*E* → *Am*^7^} > 0* despite the fact that ε*{*Am*^7^} < *ε*{*E*}.
(6) There is the phenomenon that the effect of harmony is greater than the sum of its parts [18, 60]. *ε*{*x*_1_ + *x*_2_ + *x*_3_} ≫ *ε*{*x*_1_} + *ε*{*x*_2_} + *ε*{*x*_3_}
*Apart from certain exceptions with rational intonation and octaves, the stationary tension of any combination of unique notes is observed to be larger than zero on the subharmonic plot. Hence, *${∆\hat{t}}_{\left\{x_{1}+x_{2}+x_{3}\right\}}>0$*. Likewise, the stationary tension of each note on its own is observed to be zero on the subharmonic plot. Hence, *${∆\hat{t}}_{\left\{x_{1}\right\}}=0$, ${∆\hat{t}}_{\left\{x_{2}\right\}}=0$*, and *${∆\hat{t}}_{\left\{x_{3}\right\}}=0$* for all x*_1_, *x*_2_*, and x*_3_* within musical range. Thus, by (19), ε*{*x*_1_ + *x*_2_ + *x*_3_} ≫ 0*, whereas by (20) ε*{*x*_1_} = 0, *ε*{*x*_2_} = 0, *ε*{*x*_3_} = 0*, and ε*{*x*_1_} + *ε*{*x*_2_} + *ε*{*x*_3_} = 0*. Therefore, ε*{*x*_1_ + *x*_2_ + *x*_3_} ≫ *ε*{*x*_1_} + *ε*{*x*_2_} + *ε*{*x*_3_}.

## 7. Conclusion

In this paper the notion of interharmonic and subharmonic modulations was proposed as a psychophysical basis for both stationary and transitional harmony.

In the domain of stationary harmony (tension in chord sonorities), this work presents subharmonic modulations as an integral complement to interharmonic modulations and shows how perceptual tensions [18, 36, 58, 59] and consonances [17, 19, 44] may be visualized through which.

In the domain of transitional harmony (resolution in chord progression), it unlocks the means of physically identifying, quantizing, and, thus, verifying perceptual resolutions and complications [18, 42] in acoustic features that have until now remained abstract and nontangible.

This work can be seen to bind prevailing psychoacoustic schools into a single theory. The Helmholtz school [3, 8, 12–17, 19, 20, 23] is represented by the interharmonic ∆*f* in (11). The Pythagorean school [5, 6, 11] generally seeks small values of integer *k*_*i*_ in (15) and (16) while requiring Δ*t*_*i*_ to be zero. Taking this further, if Δ*t*_*i*_ is ignored, *f*_*sub*_ in (15) would then correspond to the fusion tone in Stumpf's tonal fusion theory [3, 30]. Euler's* gradus suavitatis* [11] graded the goodness of *k*_*i*_-combinations for Δ*t*_*i*_ = 0. The adoption of 12-tone equal temperament [12, 33, 34] sought to evenly distribute interharmonic ∆*f* in (11). Since the aforementioned conditions may be generalized by a central theory of modulations across adjacent (interharmonic) and distant (subharmonic) sinusoids which stems from (3), this effectively integrates them into a general theory.

Computed values correlate strongly with perception and harmony-use statistics for both stationary (tension) and transitional (resolution) harmony.

Finally, this paper presented a psychoacoustic solution to the five fundamental questions of harmony.

## Figures and Tables

**Table 1:** Summary of correlations with consonance rankings and historical chord use.

Stationary harmony		Transitional harmony		
Dyads/intervals (2 notes)	Triads (3 notes)	Triads & tetrads (3 or 4 notes)		
		All transitions	All transitions *Excl. comp.*	Resolutions

r= 0.922	r=0.907	r= 0.903	r= 0.970	r= 0.996
p=0.0001	p=0.0000	p=0.0000	p=0.0000	p=0.0000

**Table 2:** Proposed and existing correlates of stationary harmony.

Method	Dyads	Triads
	r (p)	r (p)
*T*_Δ*f*∣Δ*t*_ * (Proposed) Equal Temperament*	0.922 (0.0000)	0.907 (0.0000)
Log Periodicity *Just *[43]	0.982 (0.0000)	0.831 (0.0002)
Rel. Periodicity *Just *[43]	0.982 (0.0000)	0.846 (0.0001)
Log Periodicity *Rational *[43]	0.936 (0.0000)	0.813 (0.0004)
Rel. Periodicity *Rational *[43]	0.936 (0.0000)	0.808 (0.0004)
Rel. Periodicity *Pythagorean *[43]	0.817 (0.0003)	-
Rel. Periodicity *Kirnberger III *[43]	0.796 (0.0006)	-
*Ω* measure [62]	0.886 (0.0000)	-
Consonance Raw Value / Degree^*∗*†^	0.978 (0.0000)	0.826 (0.0016)
Dual Process [13]^‡^	-	0.791 (0.0006)
Percentage Similarity [53]^‡^	0.977 (0.0000)	0.802 (0.0005)
Instability [18]^‡^	-	0.698 (0.0040)
Tension [18]^‡^	-	0.599 (0.0153)
Sonance Factor^$^	0.982 (0.0000)	0.434 (0.0692)
Generalized Coincidence [63]^‡^	0.841 (0.0002)	-
Consonance Value^||^	0.940 (0.0000)	0.755 (0.0014)
Dissonance Curve [21]^‡^	0.905 (0.0000)	0.723 (0.0026)
Pure Tonality [22]^‡^	0.938 (0.0000)	0.675 (0.0162)
Complex Tonality [22]^‡^	0.738 (0.0020)	-
Roughness [23]^‡^	0.967 (0.0000)	0.352 (0.1193)
Sensory Dissonance [24, 25]^‡^	-	0.607 (0.0139)
Critical Bandwidth [12]^‡^	-	0.570 (0.0210)
Temporal Dissonance [8]^‡^	-	0.503 (0.0399)
Gradus Suavitatis^#^	0.941 (0.0000)	0.690 (0.0045)

^*∗*^Raw Value was used for Dyads and Degree was used for Triads.

^†^Fotlyn, 2012, as cited in [43]

^‡^as cited in [43]

^$^Dyads from [64] and Triads from Hofmann-Engl, 2004, both as cited in [43]

^||^Brefeld, 2005, as cited in [43]

^¶^Dyads from [23] and Triads from Hutchinson & Knopoff, 1979, both as cited in [43]

^#^Euler, 1739, as cited in [43]

**Table 3:** Tabulation of $\tilde{∆∆t}$ for chords that most commonly follow V against Palestrina's chord tendencies as cited in [45].

	I	V^7^	iii^6^	V/V	V^2^	vi	V^6^/V	vi^7^	i	iii	vi^6^	I^6^	ii^6^	vii°	V^6^	ii	IV
Convergence^*∗*^	C	D	D	C	D	X↑	C	X↑	C	C	X↑	C	C	D	D	C	X↓

Frequency^†^	42	7	6	2	2	11	1	1	0	4	1	5	2	0.5	2	2	6

$\tilde{\Delta \Delta t}$	444	-1.5	1.8	1.9	0.4	3.4	2.3	1.9	6.9	1.2	0.3	39.6	1.9	7.4	4.3	2.5	192

^*∗*^States of convergence:

C denotes convergence of $∆\hat{t}$.

D denotes divergence of $∆\hat{t}$.

X↑ denotes escalating excursion of $∆\hat{t}$.

X↓ denotes descending excursion of $∆\hat{t}$.

^†^In percent, as read off the histogram of chord tendencies from [45] computed over a dataset of 11000 chords from Palestrina.

**Table 4:** Tabulation of correlations between $\tilde{∆∆t}$ and Palestrina's chord use statistics as collated in [45]. Correlations are listed in the top row with corresponding significance in brackets below.

Resolutions^*∗*^	Complications^†^	Excursions^‡^		All *excl. comp.*^ †^	All
		Escalating	Descending^§^		
0.996	-0.761	0.863	-	0.970	0.903
(0.0000)	(0.1353)	(0.3366)		(0.0000)	(0.0000)

^*∗*^Our model is designed to compute tension release in resolution.

^†^Complications in music may be interpreted as negative tension resolutions; hence, correlation seen is negative.

^‡^Excursions usually encompass tension release; however, apart from resolution alone, the perception of succeeding chords are also influenced by the rising or falling of parallel melodies.

^§^Apart from the descending excursions leading to IV, insufficient other descending transitions are recorded to compute its correlation.

## Supplementary Materials

Supplementary 1 Supplementary Figure S1: Sinusoidal Summation across 8 Amplitude Ratios. Acos⁡*ω*_1_t + Bcos⁡*ω*_2_t for various values of B normalized to A=1.

Supplementary 2 Supplementary Table S1: Correlation for Intervals. There is tabulation of ordinal ranking of dyads (intervals) using T_∆f∣∆t_ against available rankings collated in [43].

Supplementary 3 Supplementary Table S2: Correlation for Triads. There is tabulation of ordinal ranking of triads using T_∆f∣∆t_ against available rankings collated in [43].

Supplementary 4 Supplementary Audio S1: Low-Frequency Modulation. There is audio example of consonant low-frequency modulation with $\overset{-}{f}=440Hz$ and ∆*f* = 0.5*Hz*.

Supplementary 5 Supplementary Audio S2: Beating Frequency. There is audio example of dissonant high-frequency modulation with $\overset{-}{f}=440Hz$ and ∆*f* = 70*Hz*.

Supplementary 6 Supplementary Video S1: Low versus High Subharmonic Tension. There is video example comparing subharmonic wave deformation in low tension C chord against high tension Cm^7^ chord.

Supplementary 7 Supplementary Video S2: Stationary Tensions in Pachelbel's Canon. We visualize stationary harmony with subharmonic tension in Pachelbel's Canon [70].

Supplementary 8 Supplementary Video S3: Transitional Tensions in Moonlight Sonata. We visualize transitional harmony with subharmonic tension in Beethoven's Moonlight Sonata [71].
