# Supplementary figures and images for: The Science of Harmony: A Psychophysical Basis for Perceptual Tensions and Resolutions in Music
Source: Research (Wash D C). 2019 Sep 29;2019:2369041. doi: 10.34133/2019/2369041 (PMC7006947; doi:10.34133/2019/2369041)

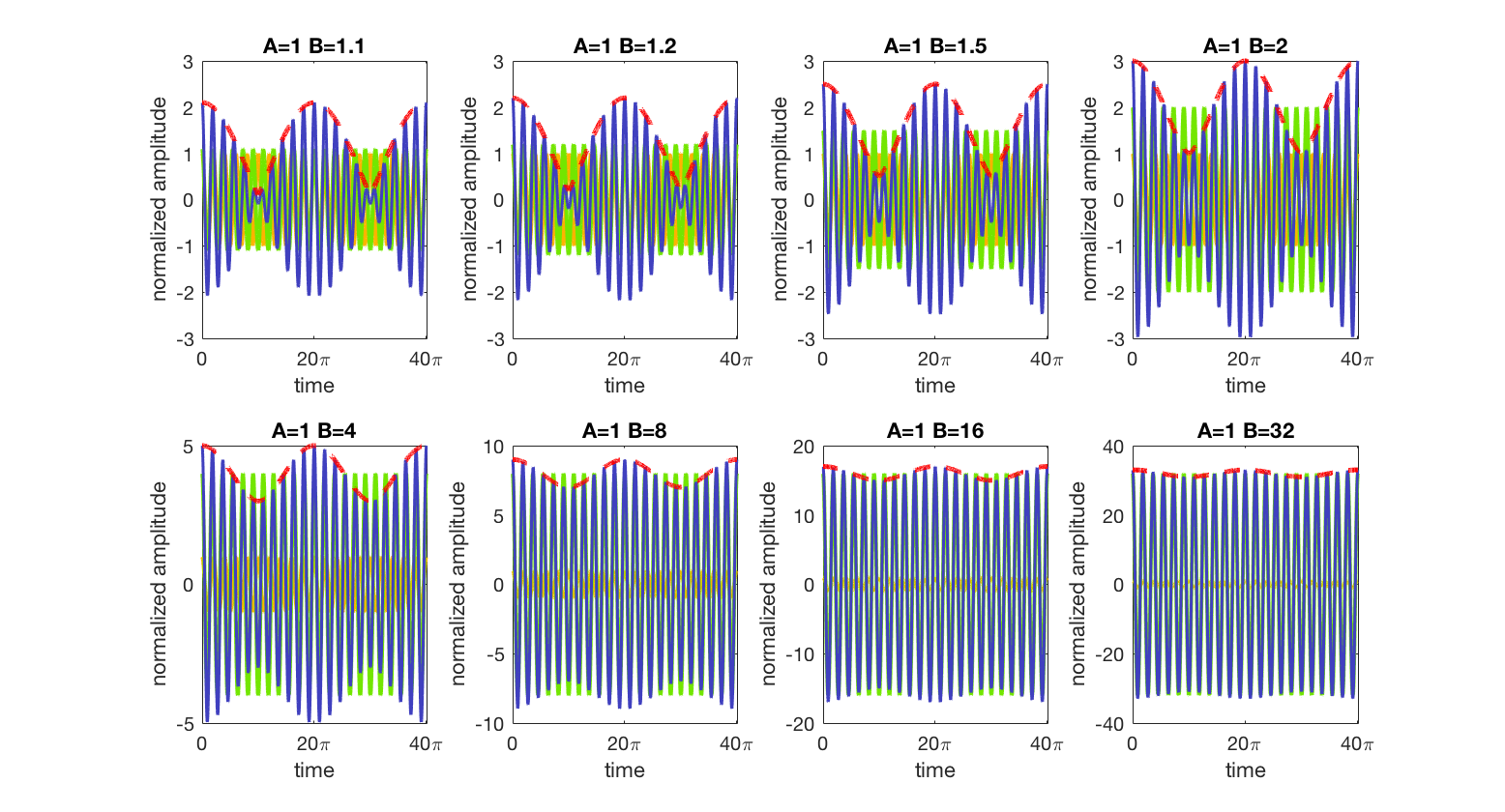


Figure S1 for various values of B normalized to A=1. Legend follows Figure 1 in text.

Supplement: Supplementary 1 — Supplementary Figure S1: Sinusoidal Summation across 8 Amplitude Ratios. Acos⁡ω1t + Bcos⁡ω2t for various values of B normalized to A=1. [file 2369041.f1.docx]
