# Supplementary material for: The Science of Harmony: A Psychophysical Basis for Perceptual Tensions and Resolutions in Music
Source: Research (Wash D C). 2019 Sep 29;2019:2369041. doi: 10.34133/2019/2369041 (PMC7006947; doi:10.34133/2019/2369041)
Supplement: Supplementary 2 — Supplementary Table S1: Correlation for Intervals. There is tabulation of ordinal ranking of dyads (intervals) using T∆f∣∆t against available rankings collated in [43]. [file 2369041.f2.docx]

| Chord | Emp. | $T_{\Delta f\vert\Delta t}$ | Rough. | Sonance | Similarity | R. Period. |
| --- | --- | --- | --- | --- | --- | --- |
| c_4_ c_4_ | 1 | 1-2 | 2 | 1-2 | 1-2 | 1-2 |
| c_4_ c_5_ | 2 | 1-2 | 1 | 1-2 | 1-2 | 1-2 |
| c_4_ g_4_ | 3 | 3 | 3 | 3 | 3 | 3 |
| c_4_ f_4_ | 4 | 4 | 4 | 4 | 4 | 4-5 |
| c_4_ e_4_ | 5 | 6 | 6 | 5 | 6 | 6 |
| c_4_ a_4_ | 6 | 5 | 5 | 6 | 5 | 4-5 |
| c_4_ a^b^_4_ | 7 | 9 | 7 | 7 | 9 | 7-8 |
| c_4_ e^b^_4_ | 8 | 8 | 10 | 8 | 7 | 7-8 |
| c_4_ f^#^_4_ | 9 | 12 | 8 | 11 | 8 | 9 |
| c_4_ b^b^_4_ | 10 | 7 | 9 | 9 | 10 | 10 |
| c_4_ d_4_ | 11 | 11 | 12 | 10 | 11 | 12 |
| c_4_ b_4_ | 12 | 10 | 11 | 12 | 12 | 11 |
| c_4_ d^b^_4_ | 13 | 13 | 13 | 13 | 13 | 13 |
| Correlation, r  Significance, p |  | 0.922  0.0000 | 0.967  0.0000 | 0.982  0.0000 | 0.977  0.0000 | 0.982  0.0000 |

Supplementary Table 1 Tabulation of ordinal ranking of dyads (intervals) using $\boldsymbol{T}_{\boldsymbol{\Delta f|\Delta t}}$ against available rankings collated in [Stolzenburg 2015].
