# Supplementary material for: The Science of Harmony: A Psychophysical Basis for Perceptual Tensions and Resolutions in Music
Source: Research (Wash D C). 2019 Sep 29;2019:2369041. doi: 10.34133/2019/2369041 (PMC7006947; doi:10.34133/2019/2369041)
Supplement: Supplementary 3 — Supplementary Table S2: Correlation for Triads. There is tabulation of ordinal ranking of triads using T∆f∣∆t against available rankings collated in [43]. [file 2369041.f3.docx]

| Class | Chord | Emp. | $T_{\Delta f\vert\Delta t}$ | Rough. | Inst. | Similarity | R. Period. | Dual Proc. |
| --- | --- | --- | --- | --- | --- | --- | --- | --- |
| Maj. | c_4_ e_4_ g_4_ | 1 | 2 | 3 | 1 | 1-2 | 2 | 2 |
|  | c_4_ e^b^_4_ a^b^_4_ | 5 | 6 | 9 | 5 | 8-9 | 3 | 1 |
|  | c_4_ f_4_ a_4_ | 3 | 1 | 1 | 4 | 5-6 | 1 | 3 |
| Min. | c_4_ e^b^_4_ g_4_ | 2 | 5 | 4 | 2 | 1-2 | 4 | 4 |
|  | c_4_ e_4_ a_4_ | 10 | 7 | 2 | 3 | 5-6 | 7 | 5 |
|  | c_4_ e_4_ a^b^_4_ | 8 | 9 | 7 | 6 | 8-9 | 10 | 6 |
| Sus. | c_4_ f_4_ g_4_ | 7 | 8 | 11 | 8 | 3-4 | 5 | 7 |
|  | c_4_ d_4_ g_4_ | 6 | 4 | 13 | 11 | 3-4 | 9 | 9 |
|  | c_4_ f_4_ b^b^_4_ | 4 | 3 | 6 | 9 | 7 | 6 | 8 |
| Dim. | c_4_ e^b^_4_ f^#^_4_ | 12 | 13 | 12 | 12 | 13 | 12 | 12 |
|  | c_4_ e^b^_4_ a_4_ | 9 | 10 | 10 | 7 | 10-11 | 11 | 10 |
|  | c_4_ f^#^_4_ e^b^_4_ | 11 | 11 | 8 | 10 | 10-11 | 8 | 11 |
| Aug. | c_4_ e_4_ g^#^_4_ | 13 | 12 | 5 | 13 | 12 | 13 | 13 |
| Correlation, r  Significance, p | |  | 0.907  0.0000 | 0.352  0.1193 | 0.698  0.0040 | 0.802  0.0005 | 0.846  0.0001 | 0.791  0.0006 |

Supplementary Table 2 Tabulation of ordinal ranking of triads using $\boldsymbol{T}_{\boldsymbol{\Delta f|\Delta t}}$ against available rankings collated in [Stolzenburg 2015].
